# Use of the Total Cancer Care System to Enrich Screening for CD30-Positive Solid Tumors for Patient Enrollment Into a Brentuximab Vedotin Clinical Trial: A Pilot Study to Evaluate Feasibility

**DOI:** 10.2196/resprot.7289

**Published:** 2017-03-20

**Authors:** Bin Li, Steven A Eschrich, Anders Berglund, Melissa Mitchell, David Fenstermacher, Hadi Danaee, Hongyue Dai, Daniel Sullivan, William L Trepicchio, William S Dalton

**Affiliations:** ^1^ Takeda Pharmaceuticals International Company Takeda Data Science Institute Cambridge, MA United States; ^2^ H Lee Moffitt Cancer Center and Research Institute Biostatistics and Bioinformatics Tampa, FL United States; ^3^ M2Gen Administration Tampa, FL United States; ^4^ MedImmune, LLC Research Bioinformatics Gaithersburg, MD United States; ^5^ Takeda Pharmaceuticals International Company Translational and Biomarker Research Cambridge, MA United States; ^6^ M2Gen Bioinformatics Tampa, FL United States; ^7^ H Lee Moffitt Cancer Center and Research Institute Blood and Marrow Transplantation Tampa, FL United States

**Keywords:** antitumor agents, CD30 antigen, clinical trial, database management systems, medical oncology

## Abstract

**Background:**

One approach to identify patients who meet specific eligibility criteria for target-based clinical trials is to use patient and tumor registries to prescreen patient populations.

**Objective:**

Here we demonstrate that the Total Cancer Care (TCC) Protocol, an ongoing, observational study, may provide a solution for rapidly identifying patients with CD30-positive tumors eligible for CD30-targeted therapies such as brentuximab vedotin.

**Methods:**

The TCC patient gene expression profiling database was retrospectively screened for CD30 gene expression determined using HuRSTA-2a520709 Affymetrix arrays (GPL15048). Banked tumor tissue samples were used to determine CD30 protein expression by semiquantitative immunohistochemistry. Statistical comparisons of Z- and H-scores were performed using R statistical software (The R Foundation), and the predictive value, accuracy, sensitivity, and specificity of CD30 gene expression versus protein expression was estimated.

**Results:**

As of March 2015, 120,887 patients have consented to the institutional review board–approved TCC Protocol. A total of 39,157 fresh frozen tumor specimens have been collected, from which over 14,000 samples have gene expression data available. CD30 RNA was expressed in a number of solid tumors; the highest median CD30 RNA expression was observed in primary tumors from lymph node, soft tissue (many sarcomas), lung, skin, and esophagus (median Z-scores 1.011, 0.399, 0.202, 0.152, and 1.011, respectively). High level CD30 gene expression significantly enriches for CD30-positive protein expression in breast, lung, skin, and ovarian cancer; accuracy ranged from 72% to 79%, sensitivity from 75% to 100%, specificity from 70% to 76%, positive predictive value from 20% to 40%, and negative predictive value from 95% to 100%.

**Conclusions:**

The TCC gene expression profiling database guided tissue selection that enriched for CD30 protein expression in a number of solid tumor types. Such an approach may improve screening efficiency for enrolling patients into biomarker-based clinical trials.

## Introduction

### Background

With the genomic age providing a greater understanding of the complexity of cancer origin and progression, the environment for cancer drug development holds great opportunity but also significant challenge [1,2]. The biopharmaceutical industry has responded by developing a treatment armamentarium that capitalizes on tumor-specific features and improves outcomes for select patient populations. These targeted therapies include small molecule inhibitors, monoclonal antibodies, antibody-drug conjugates (ADCs), and more recently, cell-based immunotherapies [2]. Despite this progress, significant challenges have emerged in the translation of genomic discoveries into clinical practice, resulting in high costs and long development times [1,3,4].

Poor clinical trial accrual rates are a leading barrier to clinical research and can impede trial efficiency [5]. Clearly, the advent of target-based therapies shows promise for identifying and selecting patients most likely to benefit from treatment; however, identifying a subset of patients who meet specific, comprehensive eligibility criteria that includes the presence of specific molecular aberrations is a significant challenge [4,6]. It is not uncommon to screen hundreds of patients over extended periods of time in order to identify a few who are phenotypically/genotypically eligible. In addition, there is often a limited window of opportunity to identify a patient that meets a trial’s eligibility criteria before their clinical status deteriorates rendering them ineligible [7]. This risk is most severe in patients with rapidly progressing diseases (metastatic disease, pancreatic or late-stage lung cancers) where treatment and survival timelines are generally very compressed. Unfortunately, these patients are often in greatest need of novel therapies. One approach to address these challenges is to use patient and tumor registries to prescreen patient populations to identify those who are phenotypically/genotypically eligible for target-based clinical trials [7]. This may allow for the identification of the most appropriate patients for treatment in an efficient manner and enhance the understanding of biomarkers found in specific tumors/patient subsets.

### CD30 and Brentuximab Vedotin

CD30, a transmembrane glycoprotein receptor, is a member of the tumor necrosis factor receptor superfamily [8-10]. While the function of CD30 has not been clearly defined, it has been implicated in both cell death and proliferation [11]. It has limited expression in healthy tissue or on resting lymphocytes; normal CD30 expression is restricted to the surface of activated T- and B-cells [12]. In contrast, CD30 is uniformly expressed on the malignant Reed-Sternberg cells of Hodgkin lymphoma (HL) and in systemic anaplastic large cell lymphoma (sALCL) [11,13]. It is also variably expressed in other types of non-HL including cutaneous T-cell lymphoma (CTCL), peripheral T-cell lymphoma, diffuse large B-cell lymphoma (DLBCL) [14,15], and some follicular lymphomas [16,17]. This expression profile makes CD30 an attractive therapeutic target for antibody-directed therapies. CD30 is also expressed in nonlymphomatous malignancies; however, the level of expression in these tumor types is variable and the prevalence of CD30 expression in these patient populations is low. CD30 expression has been reported in testicular embryonal carcinoma [18,19], multiple myeloma [20], thyroid cancer [21], lung adenocarcinoma and mesothelioma [22], mesenchymal tumors including osteosarcoma and rhabdomyosarcoma [23], undifferentiated nasopharyngeal carcinoma [24], granulocytic sarcoma [25], and acute myelogenous and mast cell leukemia [26,27]. Few data are available on CD30 protein expression across other solid tumor types.

Brentuximab vedotin (Adcetris, Seattle Genetics Inc), an anti-CD30 ADC, represents a therapeutic application that combines the target specificity of a monoclonal antibody with the cell-killing activity of a cytotoxic small-molecule drug [28]. It consists of 3 components: the anti-CD30 chimeric immunoglobulin (Ig) G1 antibody cAC10 [11,13], the microtubule-disrupting agent monomethyl auristatin E (MMAE) [13,29], and a protease-cleavable linker that covalently attaches MMAE to cAC10 [13,29,30]. Upon binding to CD30 on the surface of malignant cells, the ADC-receptor complex is internalized and traffics to the lysosome where MMAE is released by proteolytic cleavage [31]. Binding of MMAE to tubulin disrupts the microtubule network, inducing apoptotic death of the tumor cell [13]. Brentuximab vedotin is being investigated for the treatment of a variety of CD30-positive malignancies. It received accelerated approval from the US Food and Drug Administration for the treatment of HL patients who have relapsed after autologous stem cell transplant (ASCT) or after ≥2 prior multiagent chemotherapy regimens in patients ineligible for ASCT, as consolidation for HL patients at high risk of relapse or progression post-ASCT, and for the treatment of sALCL patients after failure of ≥1 prior multiagent chemotherapy regimens [32]. Brentuximab vedotin received conditional approval from the European Medicines Agency for the treatment of adult patients with relapsed/refractory (R/R) CD30-positive HL following ASCT or ≥2 prior multiagent chemotherapy regimens when ASCT or multiagent chemotherapy is not a treatment option, as consolidation for CD30-positive HL patients at increased risk of relapse or progression following ASCT, and for the treatment of R/R sALCL [33]. Approval was based on the efficacy and safety results from 2 pivotal phase 2 studies in R/R HL [34,35] and sALCL [36,37]. Brentuximab vedotin activity in solid tumors is an active area of investigation; however, due to the variable expression and low prevalence of CD30 expression in these tumors, a mechanism is required to select for CD30-positive patients that may benefit from CD30-targeted treatment.

### Objective

The Total Cancer Care (TCC) Protocol, developed at the Moffitt Cancer Center in 2006 and operationalized by M2Gen, is a personalized cancer care initiative designed to identify and address patients’ needs throughout their lifetime, including matching patients to target-based trials [38]. Here we demonstrate that the TCC Protocol may provide a solution for rapidly identifying patients with CD30-positive tumors eligible for CD30-targeted therapies such as brentuximab vedotin.

## Methods

### Total Cancer Care Protocol and Multidimensional Total Cancer Care Data Warehouse

The TCC Protocol is an ongoing, prospective, observational study used by a consortium network of 18 US hospitals that aims to recruit millions of cancer patients to deliver personalized cancer care. It requests the patient’s permission to access their entire medical history, access their tumor for research involving molecular/genomic analysis, and be recontacted in the future if a new finding is discovered that could influence their care such as eligibility for a trial. This lifetime prospective follow-up allows for the discovery and validation of biomarkers, epidemiological studies, and the development of evidence-based practice guidelines [39-41].

The consortium network sites enrolled patients into the TCC Protocol and collected tumor, blood, and urine samples. Tissue samples were snap-frozen within 15 minutes of surgical removal, shipped to M2Gen, macrodissected to ≥85% tumor purity, and quantified for the percent of malignancy, cellularity, stroma, normalcy, and necrosis. An oversight committee ensured proper access and use of tissue and that all Health Insurance Portability and Accountability Act and Human Subject Research requirements were met. The consortium network sites provided longitudinal clinical data from consented patients for integration into the multidimensional TCC Data Warehouse, a multifaceted database established to collect, relate, and interpret clinical and molecular data from all patients seen at the H Lee Moffitt Cancer Center and Research Institute and the Moffitt Cancer Center Screening and Prevention Center since 1998. Both the TCC Protocol and the TCC Data Warehouse have been previously described in detail [38].

The TCC Data Warehouse includes, but is not limited to, cancer registry data, electronic medical record data, tissue data, consent data, molecular data, imaging data, and patient self-reported data. Important to the task of matching patients to target-based clinical trials is the ability to directly query phenotypic/genotypic data residing in the Data Warehouse to perform cohort identification. A front-end tool allows investigators with a specific trial in mind to identify groups of patients based on a set of parameters, including patient inclusion/exclusion criteria and molecular signatures that define the initial patient population to be screened. Once the population has been identified and refined, physicians are notified regarding the availability of a trial and patients are evaluated for their overall suitability to participate. Investigators can also assess whether a certain trial is feasible by determining the number of patients with specific phenotypic/genotypic characteristics available to perform the trial.

### Data Extraction

The TCC Protocol was approved on January 16, 2006, by the University of South Florida Institutional Review Board and has been approved by 20 different institutional review boards across 11 states for the community hospital consortium participating in the TCC Protocol. Clinicopathological data, expression profiling, and archival tissue were extracted using deidentified linkages.

### Measurement of CD30 Gene Expression Levels

Gene expression data were generated from tumors collected from TCC-consented subjects. The TCC patient gene expression profiling database was retrospectively screened for CD30 expression. The data source was the HuRSTA-2a520709 Affymetrix arrays (GEO GPL15048). Briefly, RNA was extracted from fresh frozen tissue, amplified, and hybridized on Affymetrix GeneChips. The Affymetrix expression arrays were normalized using the Micro Array Suite 5.0 algorithm, scaled to a trimmed mean of 500 and log2 transformed. CD30 expression was extracted using the merck-NM_001243_at probeset (GPL15048). One key question to answer in the current work is what tumor types may have a clinically meaningful subset of high CD30 gene expression samples, and for this purpose the entire data set (all tissue types, primary and metastatic disease) was used to generate a global Z-score. Tumors were categorized into 3 groups: low (Z-score ≤–1), medium (–1> Z-score <1), and high (Z-score ≥1) CD30 gene expression. The distribution plot was generated in MATLAB (R2014B, The MathWorks Inc).

### Measurement of CD30 Protein Expression Levels

Banked formalin-fixed paraffin-embedded (FFPE) tumor tissue samples were provided for CD30 protein expression analysis by semiquantitative immunohistochemistry (IHC) at Quest Diagnostics (Teterboro, NJ). Briefly, tissue slides were deparaffinized using a Dako PT LINK system, washed with a Dako wash buffer, and loaded on the LINK 48 autostainer. The staining run consisted of a peroxidase block, then incubation with either a primary antibody to CD30 (clone Ber-H2 Dako) or a negative mouse IgG1 control (BD Biosciences). Visualization of the staining was performed with Dako Flex polymer-based secondary antibody and chromogen 3,3’-diaminobenzidine.

Stained slides were evaluated by board-certified pathologists at Quest Diagnostics. Based on the eligibility criteria of the phase 3 trial (NCT01578499) of brentuximab vedotin versus physician’s choice (methotrexate or bexarotene) in patients with CD30-positive CTCL [42], CD30 positivity was defined as membranous, cytoplasmic, or Golgi CD30 expression by ≥10% of either total lymphocytes or neoplastic cells at any intensity >0 on a scale of 0 to 3+. Additionally, CD30 expression was semiquantitatively determined using a composite H-score, calculated by summing the product of the percentage of cells stained (0%-100%) at each given staining intensity (0-3+). Samples were assigned to the CD30-positive protein expression group if their H-score was ≥10 or the CD30-negative protein expression group if their H-score was <10.

### Enrichment for CD30 Immunohistochemistry Staining Positivity

Statistical comparisons of Z- and H-scores were performed using R statistical software (The R Foundation), and the caret R-package was used to estimate the predictive value, accuracy, sensitivity, and specificity of CD30 gene expression.

## Results

### Patient Population

As of March 2015, the Data Warehouse had a total patient population of 426,284 patients, 120,887 of whom had consented to the TCC Protocol. In total 39,157 fresh frozen tumor specimens were collected, from which over 14,000 samples have been analyzed for gene expression (Table 1).

**Table 1:** Total number of patients enrolled in the Total Cancer Care Protocol and the type and number of molecular assays performed on tumor specimens as of March 2015.

Patients enrolled/assays performed		Number
TCC^a^Protocol as of March 2015		
	Consented patients	120,887
	Tumors/tissues collected	39,157
	Gene expression profiles	14,218
Data generated from specimens		
	CEL files^b^(gene expression data)	14,218
	Targeted exome sequencing samples	4016
	Whole exome sequencing samples	933
	Whole genome sequencing (melanoma)	13
	SNP^c^/CNV^d^(lung, breast, colon) samples with normal pairs	559
	RNA sequencing samples	696

^a^TCC: Total Cancer Care.

^b^CEL files: data files created by Affymetrix DNA microarray image analysis software.

^c^SNP: single-nucleotide polymorphism.

^d^CNV: copy-number variation.

The following steps were taken to identify patients with a solid tumor indication and high CD30 gene expression (defined as CD30 global Z-score ≥1) from the TCC Data Warehouse. Of the 120,887 patients who had consented to the TCC Protocol, 99,241 patients had an active TCC consent (not withdrawn from the TCC Protocol and site open to accrual), 49,562 patients were alive, 12,802 of whom had a tumor specimen collected as part of TCC, and gene expression profiles were available for 8307 of these patients. These gene expression profiles indicated that across solid tumor indications, 1138 patients had a CD30 Z-score ≥1 (Table 2).

**Table 2:** Identification and refinement of patient populations with a solid tumor indication with a CD30 Z-score ≥1 (n=1138) using the Total Cancer Care Data Warehouse.

TCC^a^ patients meeting the criteria	Number
Patients consented to the TCC Protocol	120,887
Patients with an active TCC consent	99,241
Patients with a vital status of “Alive”	49,562
Patients with clinical FFPE^b^ available	12,802
Patients with a CEL file^c^ to evaluate CD30 mRNA expression	8307
Patients who express CD30 with a Z-score ≥1	1138

^a^TCC: Total Cancer Care.

^b^FFPA: formalin-fixed paraffin-embedded.

^c^CEL files: data files created by Affymetrix DNA microarray image analysis software.

### CD30 Gene Expression Levels in Total Cancer Care Data Warehouse

**Figure 1:**
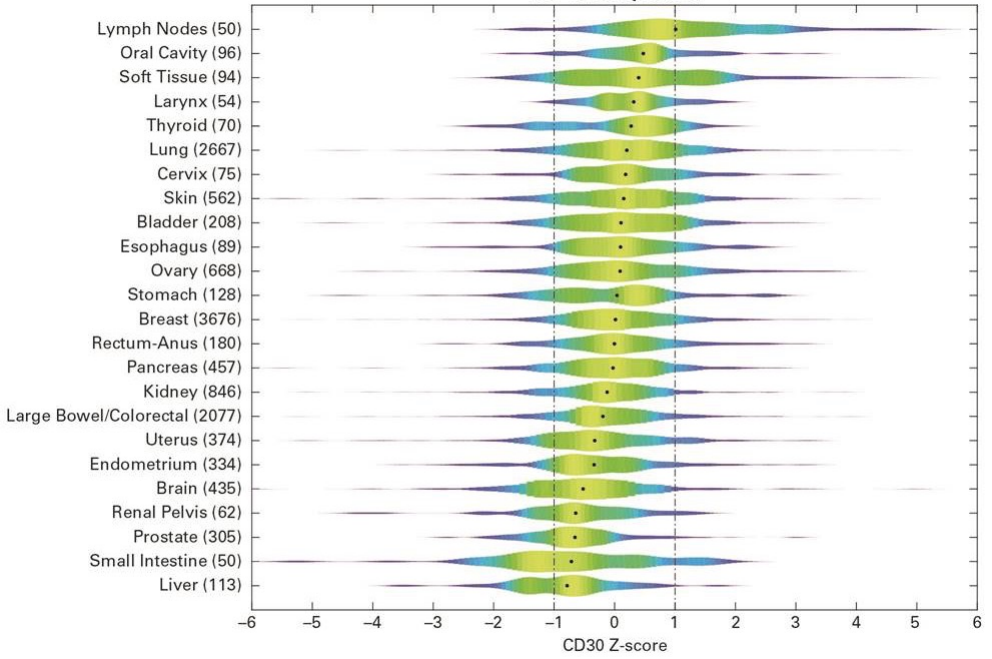
Density plot of CD30 expression Z-score across Total Cancer Care primary tumor types with ≥50 samples. The dots indicate the median value for each tissue type and the dotted lines show the Z-score cut-off.

CD30 expression across all solid tumors (n=14,218) is shown grouped by primary site in Figure 1. Aside from lymph nodes, oral cavity tumors and soft tissue (many sarcomas) show the highest median CD30 RNA expression, followed by tumors of the larynx, thyroid, lung, cervix, and skin (mostly melanoma). Table 3 lists the number of samples available, percentage of samples that tested positive (Z-score ≥1) in each group, and summarizes over or under expression of CD30. Soft tissue, skin, lung, and ovary were significantly overrepresented (*P*<.05).

**Table 3:** The enrichment of tumor site of origin for overall Z-Scores ≥1 (threshold for high-CD30 gene expression group).

Tissue type	Samples n	Samples with Z-score ≥1 n (%)	Fisher exact	Mean	Median	Standard deviation
Lymph nodes	50	25 (50)	8.12E–10	1.271	1.011	1.382
Soft tissue	94	30 (32)	5.03E–06	0.512	0.399	1.237
Ovary	668	141 (21)	6.23E–08	0.182	0.092	1.104
Oral cavity	96	20 (21)	0.051	0.444	0.475	0.887
Lung	2667	503 (19)	2.71E–17	0.178	0.202	0.986
Esophagus	89	16 (18)	0.217	0.092	0.099	0.988
Skin	562	101 (18)	0.003	0.062	0.152	1.132
Cervix	75	13 (17)	0.315	0.204	0.183	0.939
Bladder	208	35 (17)	0.185	0.087	0.107	0.996
Breast	3676	529 (14)	0.122	0.065	0.015	0.913
Rectum-anus	180	26 (14)	0.743	0.084	–0.003	0.961
Larynx	54	7 (13)	1.000	0.285	0.317	0.648
Small intestine	50	6 (12)	1.000	–0.721	–0.713	1.356
Stomach	128	15 (12)	0.606	–0.061	0.040	1.125
Uterus	374	43 (11)	0.252	–0.251	–0.329	1.036
Pancreas	457	49 (11)	0.071	–0.046	–0.026	0.940
Thyroid	70	7 (10)	0.485	0.024	0.274	0.946
Endometrium	334	30 (9)	0.012	–0.293	–0.339	0.986
Large bowel /colorectal	2077	178 (9)	1.76E–14	–0.152	–0.193	0.871
Kidney	846	53 (6)	2.72E–12	–0.154	–0.123	0.866
Brain	435	24 (6)	3.51E–08	–0.500	–0.520	1.059
Renal pelvis	62	3 (5)	0.041	–0.612	–0.643	1.022
Prostate	305	9 (3)	1.92E–10	–0.636	–0.655	0.802
Liver	113	2 (2)	2.22E–05	–0.826	–0.784	0.915

### CD30 Protein Expression Levels in Archived Tumor Samples and Enrichment for CD30 Immunohistochemistry Staining Positivity

**Figure 2:**
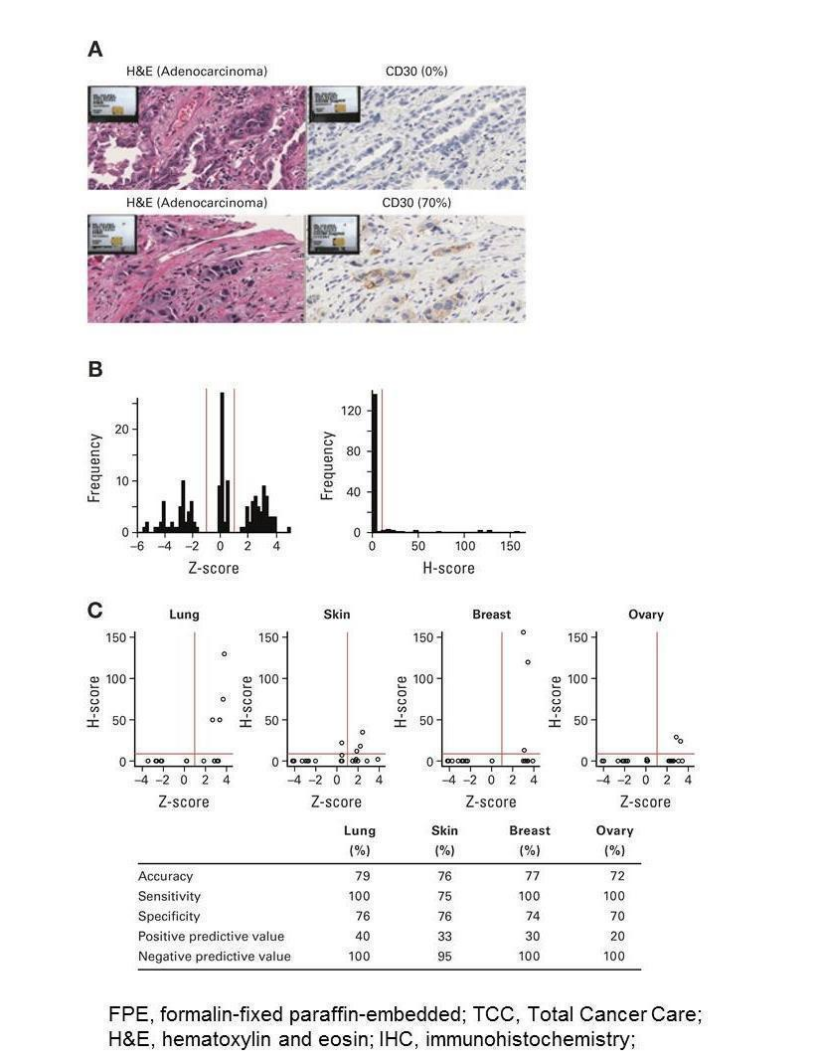
(A) Representative images of immunohistochemistry CD30 staining for 2 lung adenocarcinoma formalin-fixed paraffin-embedded slides selected based on Total Cancer Care Data Warehouse CD30 gene expression. Top: Low (0%) CD30 protein expression (H-score = 0) from medium (Z-score = 0.173) CD30 gene expression sample (ML-03-054). Bottom: High (70%) CD30 protein expression (H-score = 130) from high (Z-score = 3.784) CD30 gene expression sample (ML-03-065). (B) Distribution of gene expression Z-scores and immunohistochemistry H-scores among samples selected from the Total Cancer Care Data Warehouse. (C) Evaluation of CD30 gene expression-based enrichment of high CD30 protein expression samples.

High-level CD30 gene expression was strongly associated with IHC staining positivity. Due to the large number of tumor samples available in the TCC tissue bank for breast, large bowel, lung, skin, and ovary tumors, these tissue types were selected for confirmation of CD30 RNA expression at the protein level using IHC staining. Figure 2 A shows representative images of IHC CD30 staining for 2 lung adenocarcinoma FFPE selected based on TCC Data Warehouse CD30 gene expression. Figure 2 B shows the distribution of gene expression Z-values and IHC H-scores among all analyzed samples. For lung, skin, breast, and ovary samples, in the CD30 RNA-low group 0 out of 39 samples tested IHC-positive, in the RNA-medium group 1 out of 39 samples tested positive, and in the RNA-high group 12 out of 39 samples tested positive (Table 4). There are several reasons that may explain why only a portion of RNA-high samples (12 out of 39) tested IHC-positive. Direct estimation of CD30 expression has its limitations and this is especially true for cell-surface proteins. IHC may underreport true protein expression levels since cell surface proteins such as CD30 are continuously internalized, degraded, and reexpressed over time. It is possible that RNA could be a more sensitive measure for determining CD30 expression. A tumor sample expressing CD30 RNA but having no discernible protein expression during a discrete snapshot in time may still be able to respond to brentuximab vedotin therapy as the protein is reexpressed at the cell surface. For example, brentuximab vedotin has demonstrated significant activity in R/R DLBCL patients with variable or even apparently absent CD30 expression levels, with objective responses in 44% (17/39) and 27% (6/22) of patients, respectively [43,44]. Further studies in this area are underway. Table 4 shows that 9 or 10 random samples were chosen from RNA-low, RNA-middle, or RNA-high groups. This is an arbitrary number and is not proportional to number of samples in each group, since the goal of this work was to evaluate whether RNA expression can be used as a patient selection/enrichment strategy. All large bowel tissue samples stained negative for CD30 expression (data not shown).

Considering samples from all 4 CD30 positive tumor types, there was a significantly higher proportion of CD30 IHC-positive samples in the RNA-high group relative to low and median groups (*P*=.00018 and .0015, respectively). Across these same tumor types (lung, skin, breast, and ovary), accuracy ranged from 72% to 79%, sensitivity from 75% to 100%, specificity from 70% to 76%, positive predictive value from 20% to 40%, and negative predictive value from 95% to 100% (Figure 2 C).

**Table 4:** Enrichment of immunohistochemistry-positive samples from the CD30 RNA-high, -median and -low groups for breast, lung, skin, and ovarian cancer for the immunohistochemistry staining.

Diagnosis	Samples with RNA screened n (RNA-high)	RNA-low samples		RNA-middle samples		RNA-high samples	
		Screened n	CD30-positive n (%)	Screened n	CD30-positive n (%)	Screened n	CD30-positive n (%)
Breast	3676 (529)	10	0 (0)	10	0 (0)	10	3 (30)
Lung	2667 (503)	9	0 (0)	10	0 (0)	10	4 (40)
Ovary	668 (141)	10	0 (0)	9	0 (0)	10	2 (20)
Skin	562 (101)	10	0 (0)	10	1 (10)	9	3 (33)

## Discussion

### Principal Findings

This study reports on the utility of the TCC Protocol and demonstrates how the TCC Protocol may provide a solution for rapidly identifying patients with CD30-positive tumors eligible for treatment with the targeted therapy brentuximab vedotin. Gene expression screening was shown to enrich for protein-level CD30 expression in a number of solid tumor types, thereby demonstrating an important first filter for targeted treatment trials.

Targeted therapy is usually effective in a subset of patients with particular molecular characteristics; however, as a consequence of conducting clinical trials only in these strictly selected populations, the eligible patient pool can be low and accrual becomes more difficult [4,6]. In addition, prescreening a population of patients can be a lengthy process. Not all centers have the facilities to identify a full range of potential biomarkers, and sending tumor tissue to a central laboratory for molecular analysis to identify eligible patients can substantially extend the time between molecular analysis and treatment initiation [7]. Here we demonstrate that the TCC Protocol could help expedite clinical trials of therapies that target rare cancer populations.

In the clinical setting, patient enrollment to trials is frequently determined via protein-based IHC assay. Our results showed that CD30 expression at the RNA level significantly enriched for high CD30 protein expression and that the RNA expression level can be used as a cutoff to reliably enrich for high protein expression. Using the TCC Data Warehouse, its large volume of samples, and pregenerated large-scale expression data, we were able to identify and rank solid tumors that express CD30. CD30 is uniformly expressed in HL and sALCL [11,13]; however, few data are available on CD30 protein expression in solid tumors. Therefore a mechanism is required to select for patients with CD30-positive solid tumors that may benefit from CD30-targeted treatment. The current study indicates CD30 RNA is expressed in a number of solid tumors, and expression is highly variable, being expressed in only a subset of patients’ tumors with varying intensities. Aside from lymph nodes, oral cavity tumors and soft tissue (many sarcomas) show the highest median CD30 RNA expression, followed by tumors of the larynx, thyroid, lung, cervix, and skin (mostly melanoma). Patients with these solid tumors could serve as a baseline cohort for any subsequent subject selection for a trial where CD30 expression is used as part of the selection criteria. We acknowledge that the global Z-score cutoff used in the study may not fit well with cancer types that have very high (eg, lymph nodes) or very low (eg, liver) CD30 gene expression levels, indicating the potential future need to adjust the Z-score cutoff in individual cancer type focused clinical trials.

Tissue arrays can screen through tens to perhaps as many as several hundred samples [45]; however, the variability in technical factors and the resulting quality of tissue specimens means that the integrity of tissue samples are in many instances compromised. The TCC Protocol uses well-established, standardized processes for collecting and storing biological samples. Thousands of tumor samples can be simultaneously screened at the RNA level, providing sufficient power to identify low-prevalence markers, including CD30. Matched tumor samples collected during the same protocol period enables direct comparison between gene and protein levels within the same tumor biopsy.

IHC analysis confirmed CD30 expression at the protein level and indicates that CD30 is expressed on tumor cells and not on infiltrating lymphocytes. CD30 gene expression can therefore be considered a reliable indication of CD30 protein expression in a number of solid tumors that may benefit from treatment with a targeted CD30 therapy such as brentuximab vedotin, including breast, melanoma, ovarian, and non–small cell lung cancer. Conversely, no colorectal cancer samples (n=28) had IHC-positive CD30 protein expression (data not shown).

The TCC Protocol is unique in its approach, coordinating patient records and standardizing data collection and sample handling protocols between a wide consortium network of 18 hospitals, resulting in an extremely large reference dataset. Individual clinical studies are heavily selected for a particular study population, exclusively examining small, specialized groups, making population-level variability much harder to identify. By contrast, the TCC Protocol and the Data Warehouse captures a much larger, real-world representation of disease prevalence and may be more likely to detect gene/signature prevalence without the bias of a particular trial population.

### Conclusions

High-level CD30 gene expression can be used to significantly enrich patient populations for CD30 protein expression and could be used to guide future protein screening. The TCC Data Warehouse is not anonymized; patients are consented and enrolled into the TCC study with the full knowledge that future screening of the Data Warehouse may identify them as potential candidates for novel treatments and allows for patients to be recontacted. A uniform, large reference data warehouse like TCC may substantially increase the screening efficiency for enrolling patients into biomarker-based trials and as a consequence overcome difficulties associated with recruitment and accelerate the clinical development process. Such an approach could help to expedite clinical trials of therapies that target rare cancer populations.

## Abbreviations

ADC: antibody-drug conjugate
ASCT: autologous stem cell transplant
CNV: copy-number variation
CTCL: cutaneous T-cell lymphoma
DLBCL: diffuse large B-cell lymphoma
FFPE: formalin-fixed paraffin-embedded
H&E: hematoxylin and eosin
HL: Hodgkin lymphoma
Ig: immunoglobulin
IHC: immunohistochemistry
MMAE: microtubule-disrupting agent monomethyl auristatin E
R/R: relapsed/refractory
sALCL: systemic anaplastic large cell lymphoma
SNP: single-nucleotide polymorphism
TCC: Total Cancer Care
